# Crotoxin from *Crotalus durissus terrificus* impairs the establishment of chemically induced colitis in mice associated with formyl peptide receptor signaling

**DOI:** 10.3389/fimmu.2026.1886884

**Published:** 2026-09-16

**Authors:** Bianca de C. L. F. Távora, Sarah Natalie Cirilo Gimenes, Isabella Mitie de Camargo, Leticia Cordeiro Martins, Monica Colombini, Patricia Bianca Clissa, Sabri Saeed Sanabani, Eliana L. Faquim-Mauro

**Affiliations:** 1 Laboratory of Immunopathology, Butantan Institute, São Paulo, Brazil; 2 Department of Immunology, Institute of Biomedical Sciences, University of São Paulo, São Paulo, Brazil; 3 Laboratory of Medical Investigation LIM 03, Hospital das Clínicas (HCFMU), Faculty of Medicine, University of São Paulo, São Paulo, Brazil

**Keywords:** acute colitis, *Caco-2* and *formyl peptide receptors*, *Crotalus durissus terrificus*, crotoxin, gut microbiome, immunomodulation

## Abstract

Inflammatory bowel diseases (IBDs) arise from a breakdown in tolerance to intestinal microbiota and are characterized by exacerbated inflammatory responses. Epithelial cells of the gastrointestinal tract play a central role in maintaining barrier integrity and orchestrating immune responses; however, the mechanisms underlying epithelial dysfunction and uncontrolled inflammation remain incompletely understood. Crotoxin (CTX), the main component of *Crotalus d. terrificus* venom, exhibits immunomodulatory activity, making it a promising tool to investigate epithelial–immune interactions in intestinal inflammation. This study evaluated the effects of CTX on acute colitis induced by trinitrobenzene sulfonic acid (TNBS) in mice, as well as its ability to modulate human epithelial responses. In Caco-2 cells stimulated with IFN-γ, CTX attenuated inflammatory responses by preserving epithelial barrier integrity, maintaining ZO-1 expression and reducing ICAM-1 expression and IL-8 secretion. Furthermore, IFN-γ-stimulated Caco-2 cultures induced neutrophil and monocyte migration, an effect reduced when CTX was present. The TNBS intrarectal instillation in mice induced acute colitis, which was ameliorated by CTX administration resulting in reduced weight loss, lower clinical scores, smaller necrotic areas, decreased recruitment of neutrophils, monocytes and macrophages into the lamina propria, and a partial reversal of TNBS-induced gut dysbiosis. Notably, the protective effects of CTX were abrogated by Boc_2_, an antagonist of formyl peptide receptor (FPR), indicating the role of these receptors on the effect of CTX. In conclusion, CTX prevents early events of acute colitis by regulating epithelial and immune responses and modulating the gut microbiota through FPR-mediated pathways, highlighting its promise as a novel therapeutic candidate.

## 1 Introduction

The intestinal epithelium is crucial as the first barrier against the entry of pathogens and for the maintenance of local homeostasis. The epithelial cell layer establishes direct contact with the external environment and is continuously exposed to viruses, fungi, parasites, and potentially pathogenic bacteria (1, 2) Selective permeability of this epithelium is mediated by two distinct pathways: transcellular and paracellular. The transcellular pathway is responsible for the absorption of sugars, amino acids, peptides, fatty acids, vitamins, and other nutrients, whereas the paracellular pathway mediates the transport of molecules across the intercellular space from the intestinal lumen to the mucosal tissue and circulatory system (1, 2). Intercellular transport is regulated by apical junctional complexes, consisting of tight junctions (TJs) and adherence junctions (AJs). TJs sense stress and coordinate alterations in protein expression and assembly, thereby regulating selective permeability from the lumen to the mucosa (3). Consequently, factors that disrupt TJ expression—whether genetic or epigenetic—lead to epithelial barrier dysfunction. Such alterations have been correlated with intestinal microbiota dysbiosis and the onset and progression of inflammatory bowel diseases (IBDs) (4, 5).

Microbiota composition and factors that trigger or sustain the exacerbation of intestinal immune responses in IBD have been extensively investigated aiming new therapies (6–10). During exacerbated inflammation, the intestinal epithelium produces mediators such as IL-8, which recruit neutrophils that act against pathogens through phagocytosis and secretion of cytokines and chemokines, thereby amplifying leukocyte infiltration (11). Macrophages also participate in this process (12, 13). Proper resolution of immune cell activation is critical to terminate inflammation and prevent tissue injury (14, 15). Dysregulation of these mechanisms underlies chronic inflammatory disorders such as Crohn’s disease (CD) and ulcerative colitis (UC). Advances in IBD research have identified hyperactive mucosal signaling pathways and excessive leukocyte infiltration as key pathological features.

Conventional treatments have been focused on symptom control using corticosteroids, aminosalicylates, immunomodulators, and, in severe cases, surgical intervention. Although biologics have significantly advanced therapy, a considerable number of patients remain refractory or experience adverse effects, highlighting the need for alternative interventions. In this sense, current investigations explore novel strategies, including modulation of host–microbiome interactions, mesenchymal stem cell therapy, regulation of intestinal fibrosis, and modulation of the gut–brain axis (16, 17).

Among immunomodulatory agents, snake venoms, particularly from *Crotalus* species, have attracted interest due to their immunoregulatory potential. The venom of *Crotalus durissus terrificus* (*C.d. terrificus*) contains several bioactive components, with crotoxin (CTX) accounting for ~60% of its composition. CTX blocks neuromuscular transmission, exerts myotoxic effects at high doses, and can induce lethality depending on dose and route (18, 19). Importantly, CTX has demonstrated anti-inflammatory, immunomodulatory, antitumoral, antimicrobial, and analgesic activities (20–31). CTX is a ~24 kDa heterodimer consisting of a non-covalent acidic subunit, crotapotin (CA), devoid of toxic or enzymatic activity, and a basic phospholipase A2 (PLA2) subunit (CB) with toxic properties (32, 33). CA functions as a chaperone, guiding PLA2 to its cellular receptor, where dissociation occurs and the enzymatic activity of CB is exerted (34).

Considering its immunomodulatory potential and evidences implicating immune dysregulation in IBD, 35 evaluated CTX in TNBS-induced acute colitis in mice. CTX attenuated clinical manifestations, reduced colonic inflammation, and decreased IL-1β, TNF-α, IL-6, IL-17, Th17 cells, and ILC3 populations, while increasing TGF-β, PGE_2_, LXA_4_, and CD4^+^Foxp3^+^ regulatory T cells in the lamina propria by day 4 corresponding to the acute inflammation. Considering these data and the effective modulatory effect of CTX on the murine model of colitis we aimed to evaluate the mechanisms involved in the immunomodulation of CTX as a therapeutic potential.

### 1.1 Objective

The present study aimed to further investigate the effects of CTX in acute colitis development, specifically its modulatory potential on human intestinal epithelial cells, as well as its impact on neutrophil, monocyte, and macrophage migration during the development of acute intestinal inflammation in a murine model, and the mechanisms underlying CTX-mediated immunomodulation.

## 2 Methods

### 2.1 Animals

Male BALB/c mice with 7–8 weeks of age were bred from the animal house facilities of the Butantan Institute, (São Paulo, Brazil). The animals were kept under standard conditions of the bioterium with temperature of 25°C and humidity, 12/12 h light/dark cycle and standard feed and water *ad libitum*. All animal experiments were carried out in accordance with the ARRIVE guidelines and the Brazilian National Council for Animal Experimentation Control (CONCEA) and were approved by the Ethics Committee for Animal Studies of Butantan Institute (CEUAIB, protocol number 6206040817) and by the Institute of Biomedical Sciences, University of Sao Paulo, Brazil (protocol number 9243110419).

### 2.2 *C. d. terrificus* venom and purification of CTX

The pool of lyophilized venom was obtained from adult specimens of *Crotalus durissus terrificus* provided by the Herpetology Laboratory of the Butantan Institute. The venom was stored at –20°C until use. Purification of CTX from *C. d. terrificus* venom was performed by anion-exchange chromatography according to the method described by 36 with minor modifications. An aliquot of lyophilized rattlesnake venom (10 mg) was resuspended in 2.0 mL of Tris-HCl solution (50 mM, pH 7.3) and subsequently subjected to anion-exchange chromatography on a MONO-Q HR 5/50 column of a FPLC system (GE Healthcare), previously equilibrated with the same buffer. Proteins bound to the resin were eluted using a linear gradient of 0–1 M NaCl in 50 mM Tris-HCl, pH 7.3. After purification, fractions containing CTX were pooled, dialyzed against PBS, and their protein concentration determined by the bicinchoninic acid method (37).

### 2.3 Induction of acute intestinal inflammation and CTX administration

Firstly, mice (n = 5) were via intraperitoneally anesthetized with 100 µL a physiological solution containing ketamine (90 mg/kg) and xylazine (10 mg/kg). Acute inflammatory response was induced by intrarectal instillation of 2 mg of 2, 4, 6-trinitrobenzenesulfonic acid (TNBS) dissolved in an equal volume of 45% ethanol (38, 39). Mice received 100 µL of the TNBS solution slowly via a 3.5 F catheter connected to a 1 mL syringe (40). For induction control, a group of mice received 100 µL of 45% ethanol alone. Twelve hours after induction, the TNBS-mice groups were treated with CTX (0.045 mg/kg) or saline (control) via intraperitoneal injection (see **Supplementary Figure 1**). The CTX dose used in the *in vivo* colitis experiments was determined through preliminary toxicity assays evaluating different CTX concentrations in mice. In these initial trials, mortality was observed exclusively at a dose starting 0.170 mg/kg. Based on these findings, we selected a concentration of 0.045 mg/kg (roughly three to four times lower than the lethal threshold). Clinical evaluation of the mice was performed daily following TNBS-induction and CTX treatment, using parameters adapted from the Crohn’s Disease Activity Index (CDAI) used in patients with Crohn’s disease. Clinical scores were assigned according to observed signs: (a) weight loss (none = 0 points; 1–5% = 1 point; 5–10% = 2 points; 10–15% = 3 points; >15% = 4 points); (b) stool consistency/diarrhea (normal = 0 points; soft stools = 2 points; perianal mucus, rectal prolapse, diarrhea = 4 points). The sum of weight loss and stool consistency scores produced a CDAI ranging from 0 (unaffected) to 8 points (severe disease), adapted from Alex (41). After each period, following the kinetics of the experiments (**Supplementary Figure 1**), the animals were euthanized with an overdose of ketamine (300 mg/Kg) and xylazine (30 mg/Kg) administrated via intraperitoneal (100 µL).

### 2.4 Isolation of cell populations from the lamina propria

The lamina propria cells were isolated from the intestinal colon of mice according to Ferrer-Font (42), with modifications. The colons were collected, washed in PBS, then opened longitudinally and subsequently cut into approximately 1-cm pieces. The tissues were then incubated for pre-digestion in 10 mL of RPMI-1640 medium containing 5% FBS, 5 mM EDTA (Sigma), and 0.145 mg/mL DTT (Sigma) at 37°C under constant agitation (180 rpm) in a shaker for 20 minutes. Tissue digestion was performed through a second incubation of the colon fragments in 10 mL of RPMI-1640 medium (Invitrogen) containing 0.5 mg/mL collagenase IV (Gibco) and 0.25 mg/mL DNase I, under constant agitation (180 rpm) in a shaker at 37°C for 30 minutes. The supernatant obtained after incubation was filtered through a 70 µm cell strainer to remove debris. The resulting cells were centrifuged (1500 rpm/5 min at 4°C) and resuspended in 1 mL of RPMI-1640 medium supplemented with 3% FBS. Cell counting and viability determination were then performed using a Neubauer chamber with 0.1% trypan blue. The obtained cells were subsequently used for staining with fluorescent antibodies for flow cytometry analysis.

### 2.5 Analysis of neutrophil, monocyte, and macrophage influx in the lamina propria of different groups of mice by flow cytometry

Suspensions containing 0.5 × 10^6^ cells obtained from the intestinal colon were incubated with anti-FcγRII/RIII receptor antibody (1 µg/10^6^ cells) for 15 min at 4°C. Subsequently, 100 µL of PBS per well was added followed by centrifugation (5 min/4°C/1500 rpm), the supernatant was removed and the Fixable Viability Stain 510 reagent (FVS-510) was added diluted in PBS used to discriminate viable from non-viable cells. For the analysis of neutrophils, monocytes, and macrophages, cell suspensions were incubated with specific antibodies for each cell type (see **Supplementary Tables 1.S2**, **2.S3**). After incubation, the cells were washed and resuspended in PBS containing 0.1% paraformaldehyde (Merck). Data acquisition was performed from samples obtained from individual animals and analyzed using a FACSCanto II flow cytometer (BD) (2 × 10^4^ events).

The analysis strategy for the cellular suspensions obtained from the different groups of mice (neutrophils, monocytes, and macrophages), the first step consisted of selecting a broad gate for the total population using the parameters of granularity or internal complexity (Side Scatter Area – SSC-A, y-axis) versus relative size (Forward Scatter Area – FSC-A, x-axis) (**Supplementary Figure 4A**). Debris and cell aggregates were excluded using a dot plot of FSC-H versus FSC-A (**Supplementary Figure 4B**). The FVS-510 reagent was used to discriminate viable from non-viable cells (**Supplementary Figure 4C**). Next, the fluorescence-minus-one (FMO) methodology was applied to determine positively stained cells for the different fluorophores (43). The live cell population (FVS-510/CD45^+^), cells were further gated based on marker expression: Ly6G^+^CD11b^+^CD18^+^ for neutrophils, CD11b^+^CCR2^+^ for monocytes, and CD11b^+^MHCII^+^CD68^+^ for M1 macrophage profile (**Supplementary Figures 4D–G**). Data were analyzed using FlowJo version 10.6.2. For cytometer calibration, CompBeads (anti-rat and anti-hamster Ig κ/negative control compensation particles set, BD™) were used.

### 2.6 Histopathological analysis of the colonic portion

Individual colonic tissue samples from mice were collected for histological analysis. Colons were fixed in 4% paraformaldehyde, followed by ethanol dehydration. Samples were then embedded in paraffin and sectioned at 3–4 μm thickness. For tissue inflammation analysis, selected sections were stained with hematoxylin and eosin (H&E) and evaluated using a histopathological scoring system. Clinical parameters were assigned values based on observed signs: (a) epithelial damage (none = 0 points; minimal goblet cell loss = 1 point; extensive goblet cell loss = 2 points; minimal crypt loss with extensive goblet cell loss = 3 points; extensive crypt loss = 4 points); (b) inflammatory influx (normal = 0 points; influx near crypts = 1 point; presence of influx in mucosa = 2 points; extensive mucosal influx with edema = 3 points; submucosal infiltration = 4 points). The sum of epithelial damage and inflammatory influx scores produced an index ranging from 0 (unaffected) to 8 points (severe disease), adapted from Alex (41). To evaluate mucus-producing cells across experimental groups, tissue sections were stained with periodic acid–Schiff (PAS). Histopathological tissues were examined under a light microscope (Olympus BX51) coupled to a digital camera (Olympus DP73/cellSens imaging software), and the number of mucus-producing cells was quantified using ImageJ software.

### 2.7 Caco-2 cell culture and *in vitro* stimulation

Caco-2 human cells (1.5 × 10³ cells) were cultured in DMEM medium (Gibco, N.Y., USA) supplemented with 10% fetal bovine serum, 100 U/mL penicillin, and 100 µg/mL streptomycin, and maintained at 37°C in a humidified incubator with 5% CO_2_. Cells were seeded in 24-well plates and cultured for 17–18 days to acquire the mature intestinal epithelial cell phenotype (44). After this period, cells were either maintained in culture medium alone or stimulated with IFN-γ (40 ng/mL), CTX (250 ng/mL), or IFN-γ/CTX (40 ng/mL + 250 ng/mL) for 18 h at 37°C in a humidified 5% CO_2_ incubator (45, 46). At the end of the incubation, culture supernatants were collected for IL-8 quantification and for cell migration assays. The cells were harvested for the analysis of ICAM-1 expression by flow cytometry. All experiments were performed in triplicate and the results expressed as mean ± SEM.

### 2.8 Intestinal permeability assay of Caco-2 cells

Caco-2 human cells were seeded at a density of 1.5 × 10^4^ cells in inserts (Corning/Transwell, pore size 0.4 µm) and cultured in a humidified incubator at 37°C with 5% CO_2_ for 17 days to promote the formation of mature enterocyte-like monolayers with tight junctions. Subsequently, the culture cells were incubated with the experimental groups described in section 3.6.2. The monolayer integrity was assessed by transepithelial electrical resistance (TEER) using a Millicell Voltmeter. TEER values (Ω·cm²) were calculated using the following formula, according to the manufacturer’s instructions:

### 2.9 Analysis of ZO-1 junctional protein expression in intestinal epithelial cells by confocal immunofluorescence

After assessing monolayer integrity in the different experimental groups (Medium, IFN-γ, CTX, IFN-γ/CTX) by TEER, the inserts containing the Caco-2 cells were washed with PBS and incubated with 2% paraformaldehyde for 15 min. The inserts were then washed with PBS and incubated for 3 h in PBS containing 1% BSA and 0.2% Triton X-100. After that, the samples were washed with PBS and incubated overnight at 4°C with anti-ZO-1 antibody conjugated to the Alexa Fluor 488 fluorophore followed by washing with PBS. The inserts were excised and mounted on slides with anti-fade/DAPI. The immunofluorescence was detected using a TCS SP8 Leica Microsystems confocal microscope, and the images processed using ImageJ software (47).

### 2.10 Analysis of ICAM-1 expression in intestinal epithelial cells

Caco-2 cell suspensions were firstly incubated with anti-FcγRII/RIII antibody (1 µg/10^6^ cells) for 15 min at 4°C. Subsequently, 1 × 10^6^ cells were incubated with anti-ICAM-1-FITC antibody in PBS containing 1% FBS for 30 min at 4°C. After that, the cells were washed and resuspended in PBS containing 0.1% paraformaldehyde (Merck). All samples were prepared in triplicate and analyzed using a FACSCanto II flow cytometer (BD) with 10^4^ events acquired per sample. The results were expressed as the mean fluorescence intensity of triplicate samples ± SD.

### 2.11 IL-8 detection in Caco-2 cell supernatants

IL-8 production was evaluated using an ELISA kit according to the manufacturer’s instructions (R&D Systems). For this, supernatants of Caco-2 previously incubated with medium, with IFN-γ (40 ng/mL), CTX (250 ng/mL), or IFN-γ/CTX (40 ng/mL + 250 ng/mL) for 18 h at 37°C in a humidified 5% CO_2_ incubator were used to chemokine analysis. The IL-8 production was calculated using a standard curve generated with the recombinant protein. The results were expressed as the mean of IL-8 concentration (pg/mL) of the samples in triplicate ± SD.

### 2.12 Serum amyloid A protein quantification

The SAA production was evaluated using an ELISA kit according to the manufacturer’s instructions (Elabscience). Serum samples from mice were collected 48 and 72 hours after TNBS intrarectally instilled and treated or not with CTX. The samples were stored at –20°C for subsequent acute-phase protein analysis. SAA concentrations in the samples were calculated from a standard curve generated with recombinant protein (ng/mL). The results were expressed as the mean of SAA concentration (ng/mL) of individual samples/group ± SD.

### 2.13 Cytokine quantification in sera from mice with acute intestinal inflammation

The cytokine profiles were evaluated in the serum samples of mice from different experimental groups. A CBA kit (BD, USA) was used to assess the Th1/Th2/Th17 cytokine profiles, following the manufacturer’s instructions. Standard curves were first generated using known concentrations of recombinant cytokines. Samples were prepared by mixing 25 μL of serum with 5 μL of capture antibody for the different pro-inflammatory cytokines used in the standard curve. Samples were incubated for 1 hour at room temperature, followed by the addition of 25 μL of PE Detection Reagent Mix and a further 2-hour incubation at room temperature. Samples were then washed with 500 μL of Wash Buffer and centrifuged at 200 × g for 5 min. Supernatants were removed, and bead pellets were resuspended in 300 μL of Wash Buffer for subsequent analysis. Flow cytometry was performed using a BD Canto II cytometer, and data were analyzed with FlowJo software (BD Biosciences, CA, USA).

### 2.14 Administration of FPR antagonist in mice with acute intestinal inflammation

To evaluate the involvement of FPRs, a separate set of experiments was conducted. Acute intestinal inflammation was induced by intrarectal instillation of TNBS in groups of mice (n=5) as described in item 2.3. After 11 hours, groups of mice were treated or not with the FPR antagonist Boc_2_ (Butoxycarbonyl-2, 0.5 mg/kg) via intraperitoneal (i.p.), according to the protocol established by Nunes (48) with modifications. One hour later, the CTX (0.045 mg/kg, i.p.) or PBS were administered. The mice groups were monitored daily for weight loss and clinical signs of disease. After the acute inflammation kinetics at 24, 48, 72, and 96 hours, the mice were euthanized, and lamina propria cell suspensions were collected for flow cytometry analysis as described in item 2.4 and 2.5.

### 2.15 Isolation of human neutrophils and monocytes from peripheral blood

The experiments performed with samples of human blood were approved by the Ethics Committee (CEP-CAAE No. 28186920.8.0000.8054). Peripheral blood samples (50 mL) were collected from healthy volunteer donors at the Instituto Butantan via venipuncture using sterile disposable equipment into a solution containing 3.8% sodium citrate at a 1:10 ratio. Citrated blood samples were then layered over Histopaque 1077 at a 1:4 ratio and centrifuged at 300 × g for 30 min at 25°C. After centrifugation, the cells from the Histopaque interface were collected and counted using 0.1% trypan blue. PBMCs were subsequently sorted using a FACSAria II flow cytometer (BD). For this, PBMC suspensions were incubated for 15 min at 4°C with the antibodies listed in **Supplementary Table 3.S5**: anti-CD14-FITC (monocytes) and anti-CD16-APC/anti-CD11b-PE (neutrophils) in PBS containing 1% FBS. After that, the cells were then resuspended and subjected to cell sorting. Monocyte- and neutrophil-positive cells were collected for use in the *in vitro* transwell migration assay. Human neutrophils (2 × 10^5^) and monocytes (1 × 10^5^) were placed in transwell systems using polycarbonate membranes (6.5 mm diameter; 3 µm pores for neutrophils, 8 µm pores for monocytes). The lower chambers were filled with supernatants from Caco-2 cells previously incubated for 18 h with Medium, CTX, IFN-γ, and CTX/IFN-γ. The transwells were incubated for 3 h at 37°C in a humidified 5% CO_2_ incubator. The inserts were stained using the May-Grünwald–Giemsa method, according to Imbert & Khoury (49) with modifications. Then, the membranes were excised, mounted on slides, and migrated cells were counted using the methodology of Leavesley (50). The results are expressed as mean ± SEM from three independent experiments.

### 2.16 Fecal microbiota analysis by 16S rRNA gene sequencing

Microbial community profiling was conducted on rectal fecal samples collected from five mice per experimental group: Naïve, Ethanol control, TNBS-induced acute colitis, and TNBS-induced colitis treated with CTX. Samples were collected 24 hours after TNBS intrarectal instillation and subsequent CTX treatment. Approximately one gram of stool per mouse was collected directly into 6iagen PowerFecal bead tubes, homogenized, and stored at -80°C. Bacterial DNA was extracted using the QIAamp PowerFecal Pro DNA kit (QIAGEN). The V3-V4 hypervariable regions of the 16S rRNA gene were amplified using the Bact_341F/Bact_805R primer set (PMID: 38267511). The resulting amplicons were purified, indexed, and sequenced on an Illumina MiSeq platform with 300 bp paired-end reads. After quality control filtering, sequence data were processed using EzBioCloud’s 16S-based Microbiome Taxonomic Profiling (MTP) pipeline. Unique reads were dereplicated and clustered into operational taxonomic units (OTUs) against the EzBioCloud 16S rRNA database. Compositional analysis of gut bacteria was performed at the phylum and genus levels.

### 2.17 Statistical analysis

Experiments were performed in at least three independent biological replicates, and data were presented as the mean ± standard deviation of the mean (SD). Statistical analyses were conducted using GraphPad Prism 10.6.1 software (GraphPad Software, Inc., San Diego, CA, USA). Comparisons among three or more groups were analyzed by one-way analysis of variance (One-Way ANOVA), followed by Tukey’s multiple-comparisons *post hoc* test when appropriate. Experiments involving two independent variables were analyzed using two-way analysis of variance (Two-Way ANOVA), followed by Tukey’s multiple-comparisons *post hoc* test. Differences were considered statistically significant when *p* < 0.05, with significance levels indicated as follows: *p* < 0.05 (*), p < 0.01 (**), *p< 0.001* (***) and *p < 0.0001* (****).

## 3 Results

### 3.1 CTX administered *in vivo* improves clinical outcomes in mice with acute colitis

To evaluate the protective effect of CTX on the establishment of acute inflammatory disease, we previously confirmed the effects observed by the administration of CTX after TNBS-instillation in mice. The **Figures 1A, B** shows that CTX reduced the weight and clinical score in TNBS-mice group. The results confirmed that CTX was able to attenuate these parameters of acute colitis in mice, as reported by Almeida (35). In addition, **Figure 1C** shows the colon of a mouse/each experimental group, allowing to observe an evident necrotic area in the TNBS-induced colitis.

**Figure 1:**
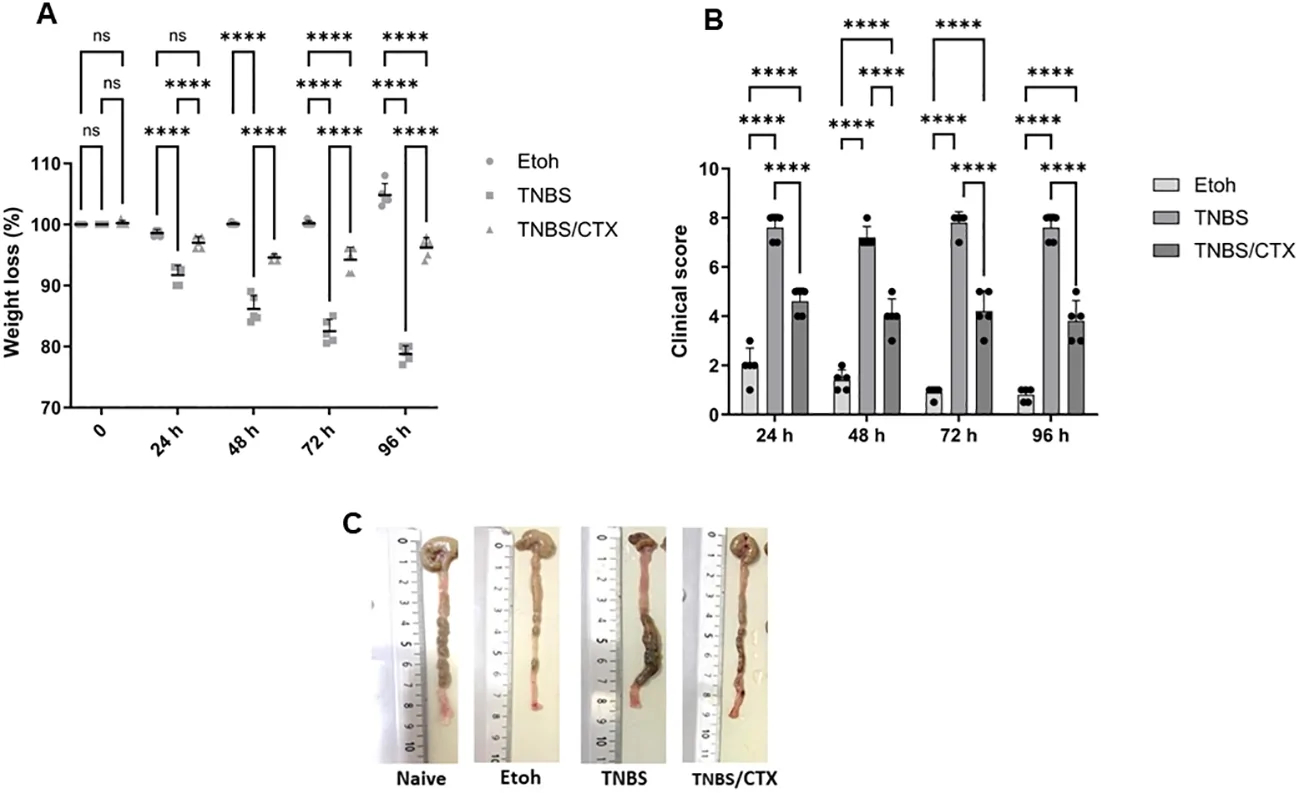
Effect of CTX on weight loss and clinical score in BALB/c mice after acute colitis induction. **(A)** Percentage of weight loss in BALB/c mice (n = 5) after acute colitis induction. CTX was administered 12 hours after acute colitis induction, and animals were evaluated at 24, 48, 72, and 96 hours following intrarectal TNBS instillation. **(B)** Clinical score corresponding to the sum of points for weight loss and stool consistency for each animal. **(C)** Macroscopic evaluation of the colonic portion of BALB/c mice after acute colitis induction. Results expressed as the mean of individual mice/group ± SD, ****p < 0.0001; ns, not significant.

### 3.2 CTX restores goblet cells during TNBS-induced colitis

Goblet cells are essential for maintaining intestinal epithelial barrier function during homeostasis, and their impairment is correlated with IBD development (51). Therefore, we evaluated the effect of CTX on mucus-producing cells in the colonic epithelium of TNBS-mice group. As shown in **Figure 2C**, TNBS-mice exhibited a decrease in mucus-producing cells compared to the Etoh group. In contrast, CTX treatment was able to impair the TNBS effect on goblet cells (**Figures 2C, D**).

**Figure 2:**
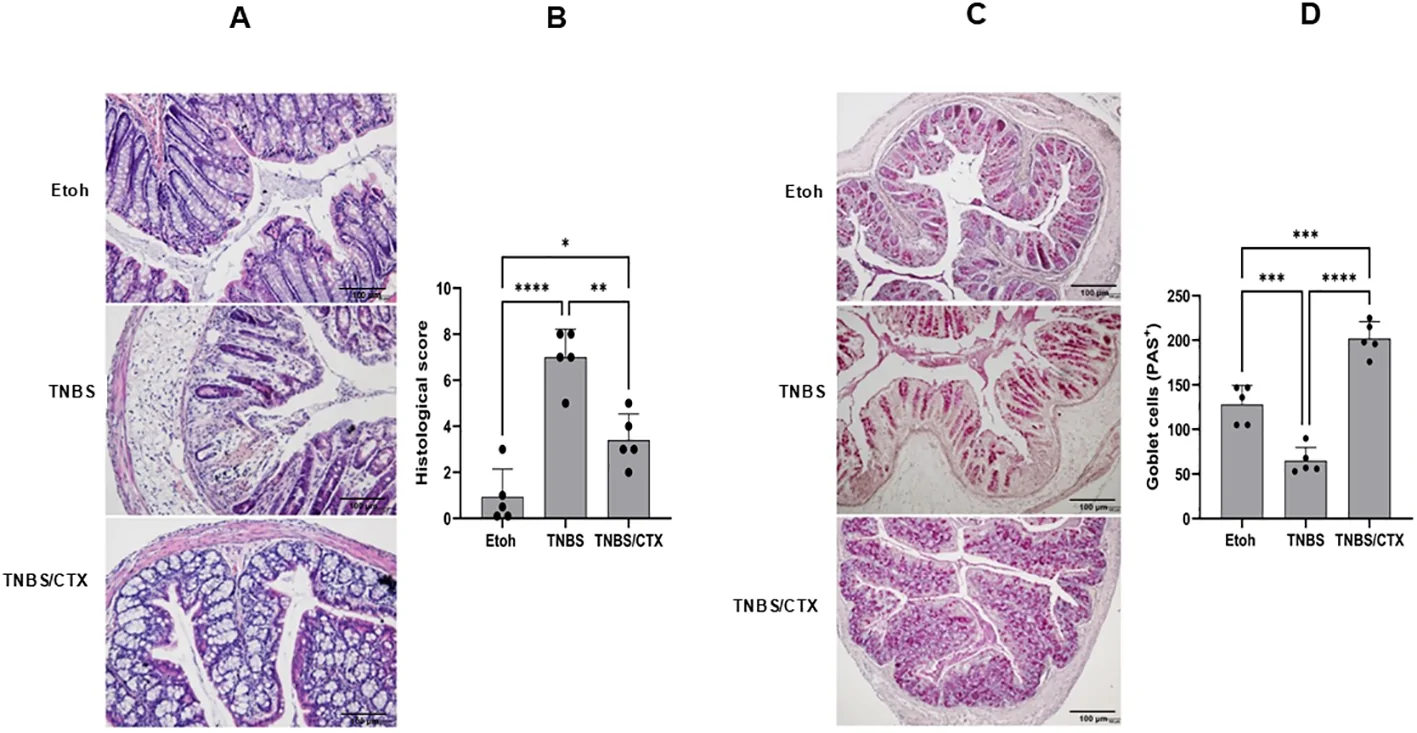
Histological analysis of BALB/c mice after acute colitis induction and CTX treatment. Colitis was induced by TNBS-intrarectal instillation in mice (n = 5). Mice received CTX (0.045 mg/kg, i.p.) or PBS 12 h later. Colons were collected 48 h post-induction. **(A)** H&E staining of colonic tissue. **(B)** Inflammation scores (mean ± SD, 20×). **(C)** Goblet cells detected by PAS staining. **(D)** Quantification of PAS-positive cells using ImageJ (mean ± SD, 10×). Results expressed as the mean of individual mice/group ± SD, *p < 0.05; **p < 0.01; ***p < 0.001; ****p < 0.0001; ns, not significant.

As previously reported by Thoo and collaborators (52), intestinal inflammation induces damage to the epithelial barrier, followed by an increased influx of inflammatory cells. Therefore, we evaluated whether the CTX would be able to prevent the breakdown of the intestinal barrier after TNBS-intrarectal instillation in mice. The histological analysis of the colonic tissue from 48 hours after TNBS-instillation showed that the CTX administration in TNBS-group inhibited the extensive infiltration of inflammatory cells induced by TNBS instillation (**Figures 2A, B**).

### 3.3 CTX modulates the integrity of the intestinal epithelial barrier in Caco-2 cells

Altered intestinal mucosal permeability is a major manifestation of barrier dysfunction, leading to disruption of intercellular tight junctions (TJs) and contributing to the development of colitis (3). In this context, we evaluated the direct effect of CTX on the barrier integrity of Caco-2 cells stimulated *in vitro* with the pro-inflammatory cytokine IFN-γ. Differentiated Caco-2 monolayers were maintained in culture medium or incubated with IFN-γ, CTX, or IFN-γ/CTX for 18 hours. Transepithelial electrical resistance (TEER) was then measured. The results showed that IFN-γ significantly decreased TEER in Caco-2 cultures compared to cells maintained in DMEM or incubated with CTX alone. However, CTX was able to prevent the IFN-γ-induced decrease in TEER, suggesting a modulatory effect of CTX on intestinal epithelial barrier integrity (**Figure 3A**). ZO-1 (zonula occludens-1) is essential for coordinating TJ formation and establishing cell polarization, mediating interactions between epithelial cells (53). Moreover, we analyzed ZO-1 expression in Caco-2 monolayers under different experimental conditions by immunofluorescence. The **Figures 3B, C** shows a reduction of ZO-1 expression in Caco-2 monolayer culture incubated with IFN-γ. In contrast, CTX modulated the effect of IFN-γ on ZO-1 expression by Caco-2 cells, presenting similar results observed in cells maintained in culture with medium or CTX.

**Figure 3:**
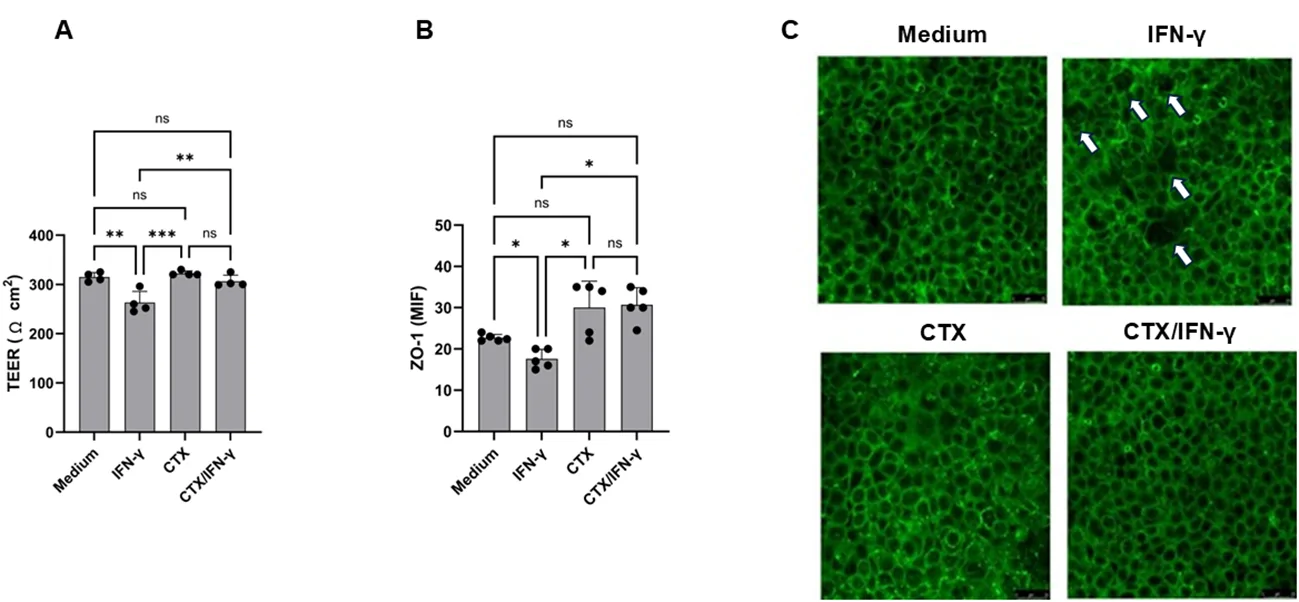
Analysis of the integrity of differentiated Caco-2 monolayers *in vitro* incubated with IFN-γ in the presence or absence of CTX. Caco-2 cells were stimulated with IFN-γ (40 ng/mL), CTX (250 ng/mL), or IFN-γ/CTX (40 ng/mL + 250 ng/mL) for 18 hours. **(A)** Transepithelial Electrical Resistance (TEER, Ω) was measured using a Millicell Voltmeter – ERS (Merck-Millipore). Results are expressed as the mean (Ω·cm²) of quadruplicate samples ± SD. **(B)** Caco-2 cells were processed and incubated with anti-ZO-1 antibody conjugated to Alexa Fluor 488 and analyzed using a Leica confocal microscope (60× magnification). Results are expressed as the mean fluorescence intensity (MFI) of quintuplicate samples ± SD. Representative results from three independent experiments are shown. **(C)** Representative image of Caco-2 cells stained with anti-ZO-1 antibody conjugated to Alexa Fluor 488. Results expressed as the mean of individual mice/group ± SD, *p < 0.05; **p < 0.01; ***p < 0.001; not significant.

### 3.4 CTX modulates serum amyloid A production in mice with acute colitis

Increased SAA production has been correlated with several acute inflammatory events. Its activity is associated with the activation of receptors on neutrophils and monocytes, as well as the enhanced production of pro-inflammatory cytokines triggering the tissue damage (54). Considering this, we investigated the SAA release in serum of TNBS-mice that were treated or not with CTX. Increased levels of SAA in samples of TNBS-mice compared with control group (Etoh) at 48 hours, as showed in **Figure 4**. In contrast, a lower production of SAA was observed in TNBS-mice treated with CTX compared with TNBS-group. At 72 hours post-colitis induction, SAA levels were decreased in all groups, with no significant differences between them. These results suggested an important modulatory effect of CTX on SAA production occurring within the first 48 hours post-TNBS colitis induction.

**Figure 4:**
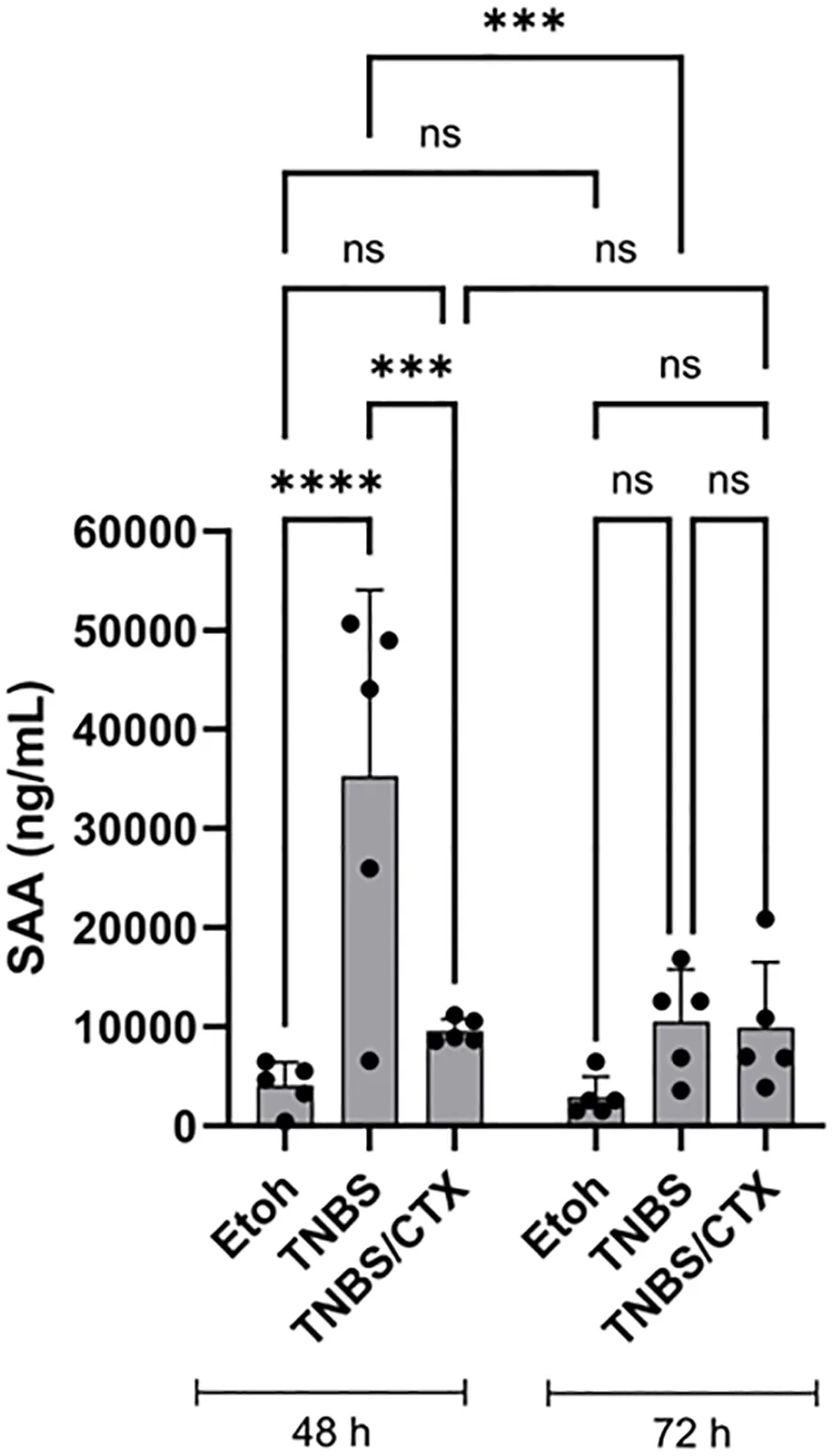
Effect of CTX on serum amyloid A (SAA) production in the serum of BALB/c mice with acute colitis treated or not with CTX. BALB/c mice (n = 5) were intrarectally instilled with TNBS and treated or not with CTX. At 48 and 72 hours after acute colitis induction, the mice were euthanized, and blood was collected by cardiac puncture to measure SAA levels in the serum samples using ELISA. Results expressed as the mean of individual mice/group ± SD, ***p < 0.001; ****p < 0.0001 ns, not significant.

### 3.5 Effect of CTX on pro-inflammatory cytokine production in mice with acute colitis

Pro-inflammatory cytokines are produced in high amounts during intestinal inflammation, resulting in an exacerbated inflammatory response (55). Therefore, we analyzed the cytokine levels (IL-1β, IL-2, IL-4, IL-6, IFN-γ, TNF-α, IL-17A, and IL-10) in serum samples obtained from the experimental groups after 24, 48, 72, and 96 hours of TNBS instillation. Mice with colitis exhibited significantly elevated production of IL-6 and TNF-α compared to control mice at all analyzed time points (**Figure 5**). Furthermore, CTX treatment attenuated the production of these cytokines, although this difference was not statistically significant for TNF-α. IL-1β, IL-2, IL-4, IFN-γ, IL-17A, and IL-10 levels were below the detection limit (data not shown). **Figure 5B** shows significant modulatory effect of CTX on IL-6 production in TNBS-mice group at 72 hours. Consistent with these findings, high levels of IL-6 detected in the serum and intestinal mucosa of patients with active Crohn’s disease positively correlate with clinical activity, as well as with the severity of endoscopic and histopathological signs (56). The inhibition of IL-6 production verified in serum of TNBS-mice that received CTX is in agreement with our data showing lower weight loss and clinical score and areas of necrosis in these mice.

**Figure 5:**
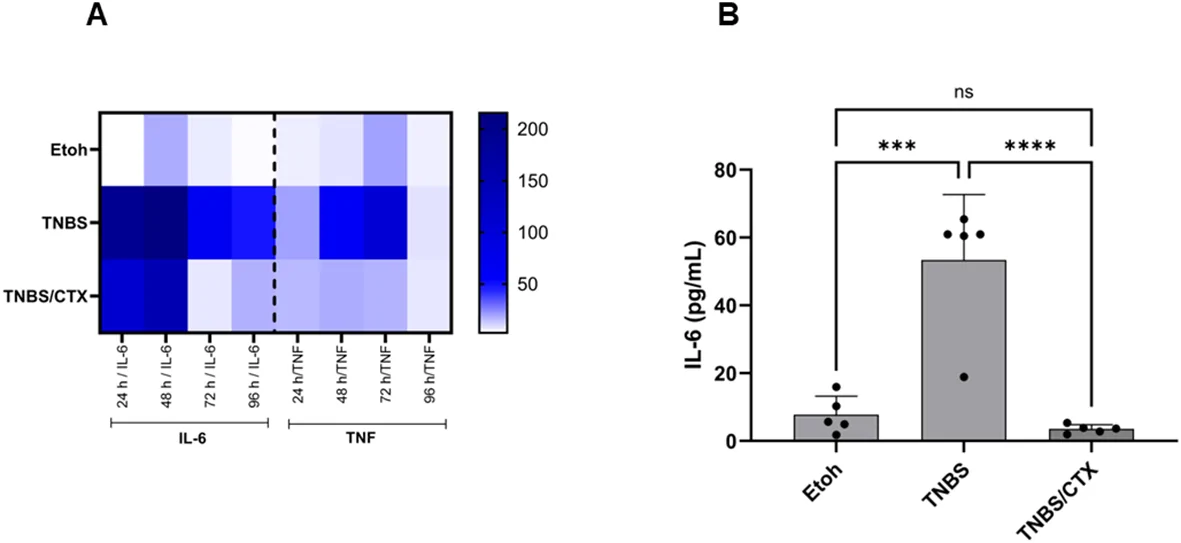
Effect of CTX on pro-inflammatory cytokine production in the serum of BALB/c mice with acute TNBS-induced treated or not with CTX. BALB/c mice (n= 5) were intrarectally instilled with TNBS and treated or not with CTX. At 24, 48, 72, and 96 hours, animals were euthanized, and blood was collected via cardiac puncture for the measurement of pro-inflammatory cytokines using the CBA method. Cytokine concentrations are expressed as pg/mL. **(A)** Heatmap of IL-6 and TNF-α production (results expressed as cytokine concentration directly proportional to the intensity of the blue). **(B)** IL-6 production at 72 hours after TNBS-instillation. Results expressed as the mean of individual mice/group ± SD, ***p < 0.001; ****p < 0.0001; ns, not significant.

### 3.6 CTX treatment partially reverses TNBS-induced gut dysbiosis

The dysfunctional interaction between the intestinal microbiota and the mucosal immune system can disrupt immune tolerance, triggering exacerbated inflammatory response. In intestinal inflammation, dysbiosis is characterized by reduced microbial diversity. Furthermore, it has been shown that the microbiota can modulate and ameliorate the intestinal inflammation symptoms (57). Accordingly, we investigated the potential of CTX to modulate microbiota in TNBS colitis mice. Analysis of the gut microbiome at 24 hours revealed a significant change in bacterial composition following TNBS instillation (**Figure 6**). At the phylum level, the TNBS group showed a marked shift compared to both naive and Etoh controls, with a substantial increase in the relative abundance of Proteobacteria and a corresponding decrease in Firmicutes and Bacteroidetes. At 24 hours after TNBS-colitis, the treatment with CTX partially reversed this dysbiosis, reducing Proteobacteria and increasing Firmicutes and Bacteroidetes populations.

**Figure 6:**
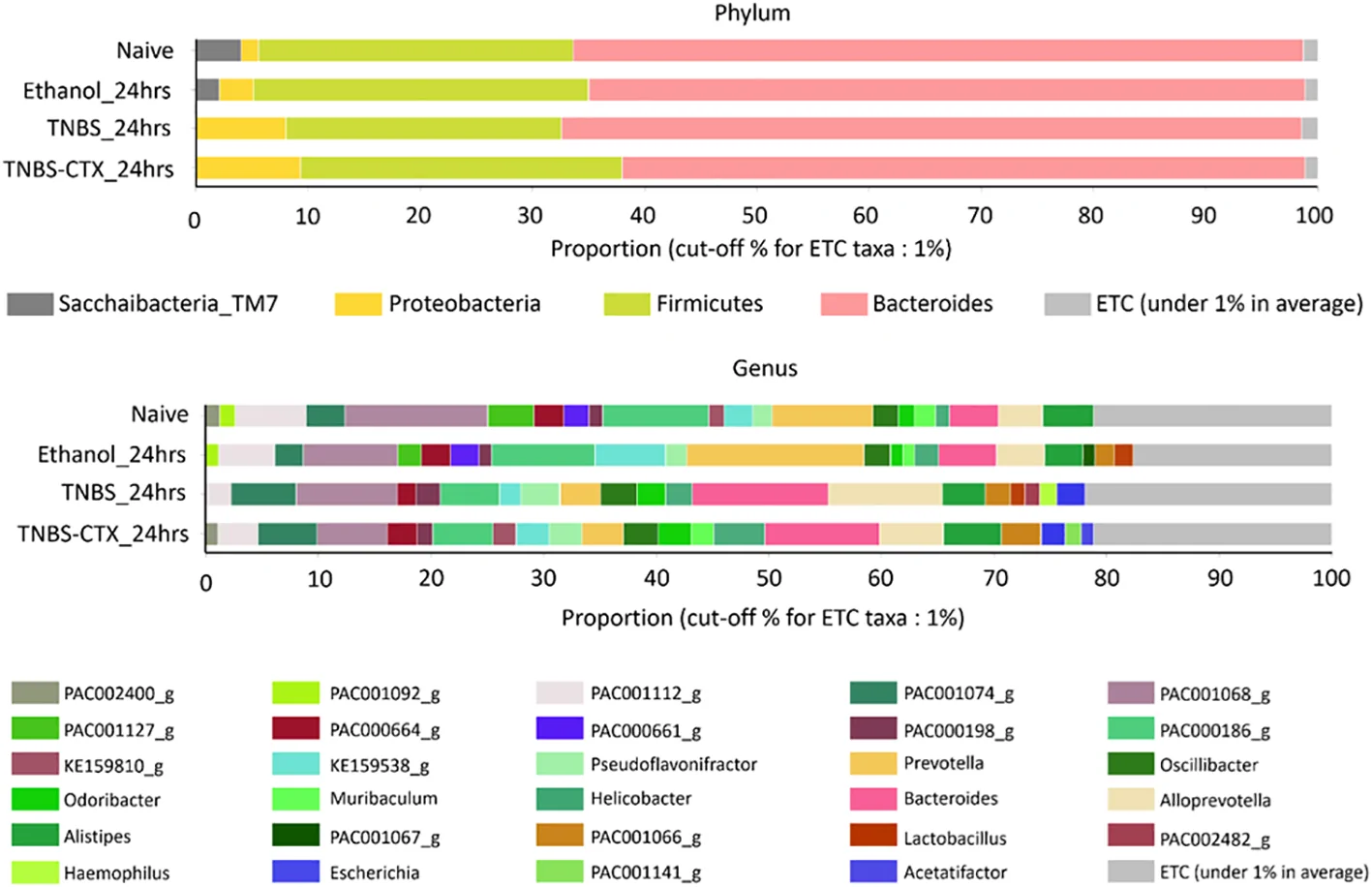
CTX treatment partially reverses gut microbiota dysbiosis 24 hours after TNBS-induced acute colitis. Relative abundance of bacterial communities in fecal samples from Naïve, Ethanol, TNBS, and TNBS-CTX treated BALB/c mice (n = 5 per group). (Top panel) Composition at the phylum level shows Proteobacteria in the TNBS group, which is reduced by CTX treatment. (Bottom panel) Composition at the genus level shows that the increase in Proteobacteria is largely driven by *Escherichia*. Taxa with an average abundance below 1% are grouped as “ETC (under 1% on average)”.

At the genus level, the increase in Proteobacteria in TNBS group was primarily due to an expansion of Escherichia, along with a reduction in several commensal genera. Administration of CTX in TNBS group was associated with a notable decrease in the relative abundance of Escherichia and a restored presence of other key genera, indicating a shift back toward the microbial community structure observed in the control groups.

### 3.7 FPR blockade impairs the modulatory effect of CTX in acute colitis

The role of FPRs in the immunomodulatory activity of CTX has been described in distinct experimental models (48, 58–60). Therefore, we treated mice with a selective FPR inhibitor (Boc_2_) and evaluated the modulatory effect of CTX in TNBS-mice. As shown in **Figures 7A, B**, CTX attenuated weight loss and the disease clinical score in TNBS-induced mice. Conversely, FPR blockade abolished the modulatory effects of CTX on these clinical parameters (**Figures 7C, D**). Taken together, these findings demonstrate that CTX action is associated with FPR signaling.

**Figure 7:**
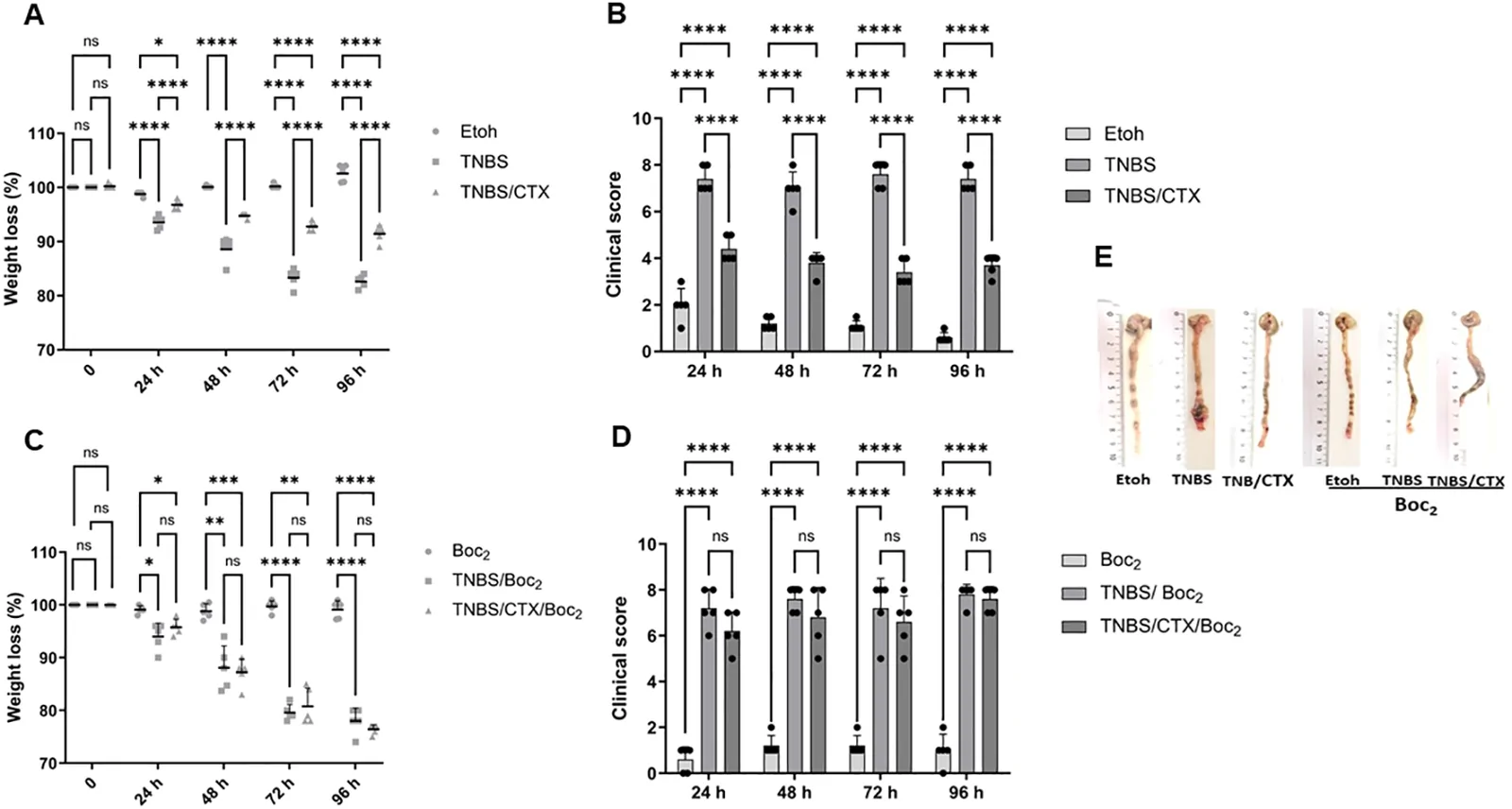
Effect of CTX on weight loss and clinical score in mice with acute colitis treated or not with Boc_2_. **(A)** Assessment of weight loss (%) and **(B)** clinical score in BALB/c mice (n = 5) induced by intrarectal instillation of TNBS and treated or not with CTX 12 h later. **(C)** Assessment of weight loss (%) and **(D)** clinical score in BALB/c mice (n = 5) induced by intrarectal instillation of TNBS and, after 11 h, received the FPR antagonist Boc_2_ (0.5 mg/kg). After 1 h, mice were treated or not with CTX (0.045 mg/kg) or PBS via i.p. **(E)** Macroscopic analysis of the colons of the animals. Results expressed as the mean of individual mice/group ± SD, *p < 0.05; **p < 0.01; ***p < 0.001; ****p < 0.0001; ns, not significant.

### 3.8 The modulatory effect of CTX on mucus production and colonic epithelial in mice with acute intestinal inflammation is FPR-mediated

The histological analysis of colonic sections from TNBS-mice treated or not with Boc_2_ showed loss of goblet cells and therefore, changes in mucus producing cells, compared to the Etoh-groups (**Figure 8**). Furthermore, the blockage of the FPRs (Boc_2_) significantly abolished the modulatory effect of CTX in the histological damage and goblet cells, indicating that the modulatory effect of CTX is mediated by this pathway (**Figure 8**).

**Figure 8:**
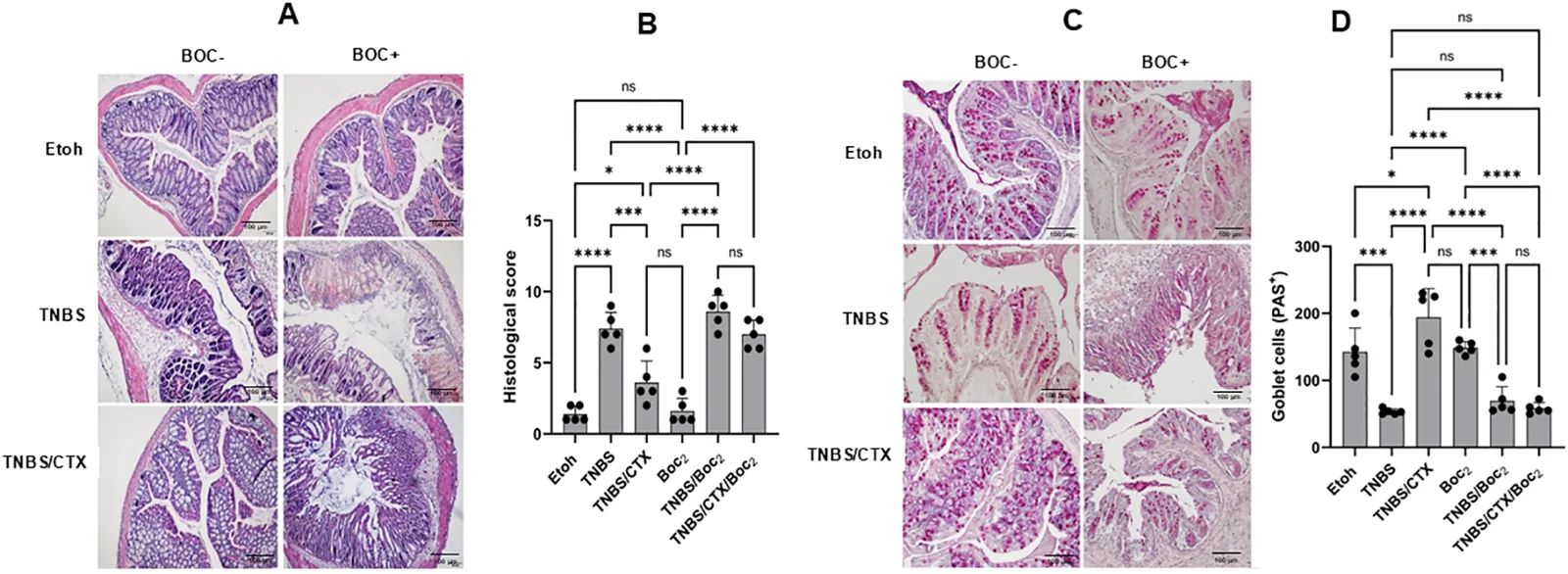
Histological analysis of colonic portion of mice with acute colitis, treated or not with Boc_2_ and receiving or not CTX. Groups of mice (n = 5) received intrarectal instillation of TNBS or Etoh. After 11 hours, they were administered or not the selective FPR antagonist Boc_2_ (0.5 mg/kg, i.p.). One hour later, the mice received or not CTX (0.045 mg/kg, i.p.). Forty-eight hours after acute colitis induction, the colonic tissue was collected and processed. **(A)** Representative images of colonic tissue inflammation stained with hematoxylin-eosin. **(B)** Histological scoring of intestinal tissue inflammation. **(C)** Representative images of goblet cells in colonic samples were identified by Periodic Acid–Schiff (PAS) staining. **(D)** Quantification of PAS-positive cells per crypt was performed using ImageJ software. Results expressed as the mean of individual mice/group ± SD, *p < 0.05; ***p < 0.001; ****p < 0.0001; ns, not significant.

Consistent with the diminished goblet cell number (PAS^+^), marked damage of intestinal epithelium accompanied by increased inflammatory cell infiltration were observed in the TNBS group, regardless of Boc_2_ treatment, when compared to the Etoh and CTX-TNBS groups. Additionally, Boc_2_ treatment abolished the modulatory effect of CTX on colonic epithelial damage in TNBS-induced mice, as showed in **Figures 8C, D**. Consequently, these results indicate that the protective effect of CTX on colonic epithelial integrity is FPR-mediated.

### 3.9 FPR blockade abrogates the effect of CTX on cell recruitment

The kinetic of neutrophil, monocyte and macrophage migration to lamina propria in TNBS-colitis mice that received or not the CTX was evaluated, as well as the role of FPR in this process. The **Figure 9** shows the results obtained in experimental groups at 24, 48, 72 and 96h of TNBS colitis induction. The data demonstrated significant increase of neutrophils (Ly6G^+^CD11b^+^CD18^+^) in TNBS-group compared with the Etoh-group in all time points. The CTX exerted a significant inhibition in the neutrophil migration in TNBS-mice group, however the Boc_2_ treatment abolished this modulatory effect.

**Figure 9:**
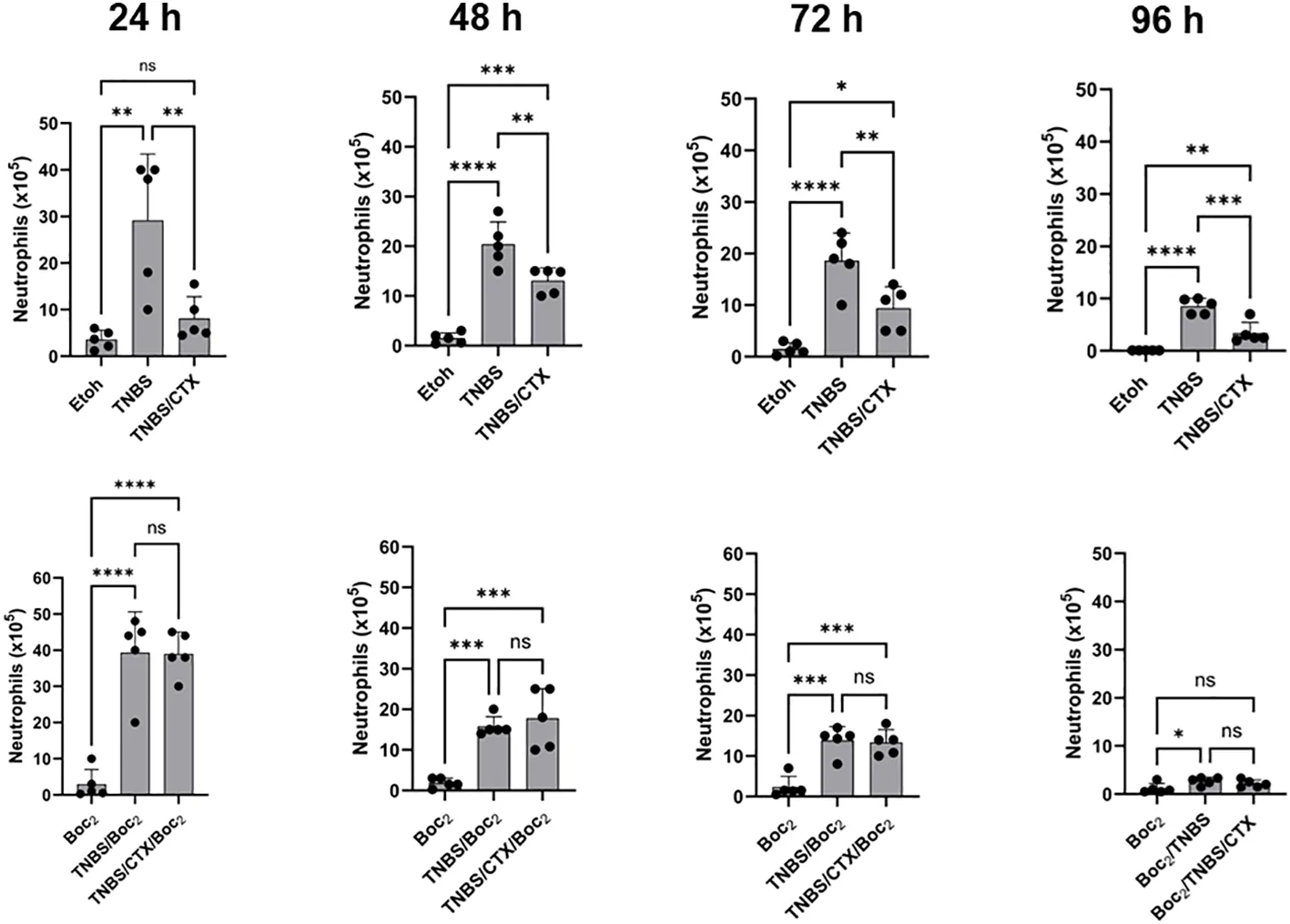
Effect of CTX on neutrophil influx in BALB/c mice after acute colitis induction treated or not with Boc_2_. Mice (n=5) received 100 µL of the TNBS solution intrarectal via. For induction control, a group of mice received 100 µL of 45% ethanol alone. Twelve hours after induction, the TNBS-mice groups were treated with CTX (0.045 mg/kg) or PBS (control) via i.p. injection. In a different set of experiments, groups of mice (n = 5) received intrarectal instillation of TNBS or Etoh. After 11 h, they received or did not receive an i.p. injection of the selective FPR antagonist Boc_2_ (0.5 mg/kg). One hour later, animals received or did not receive CTX. At 24, 48, 72, and 96 h post-colitis induction, cell suspensions from the lamina propria were incubated with fluorescent antibodies: anti-Ly6G, anti-CD11b, and anti-CD18, to analyze neutrophil influx by flow cytometry. Results expressed as the mean absolute number of positively labeled cells per animal/group ± SD. *p < 0.05; **p < 0.01; ***p < 0.001; ****p < 0.0001; ns, not significant.

Regarding monocytes, CTX reduced the influx of the CD11b^+^CCR2^+^ population at 24–72 h compared to the TNBS group (**Figure 10**). Furthermore, FPR blockade completely prevented the CTX effect as verified in Boc_2_/TNBS/CTX group, with similar monocyte influx to those of the untreated TNBS group. These finding also reinforce that CTX acts in an FPR-mediated manner (**Figure 10**).

**Figure 10:**
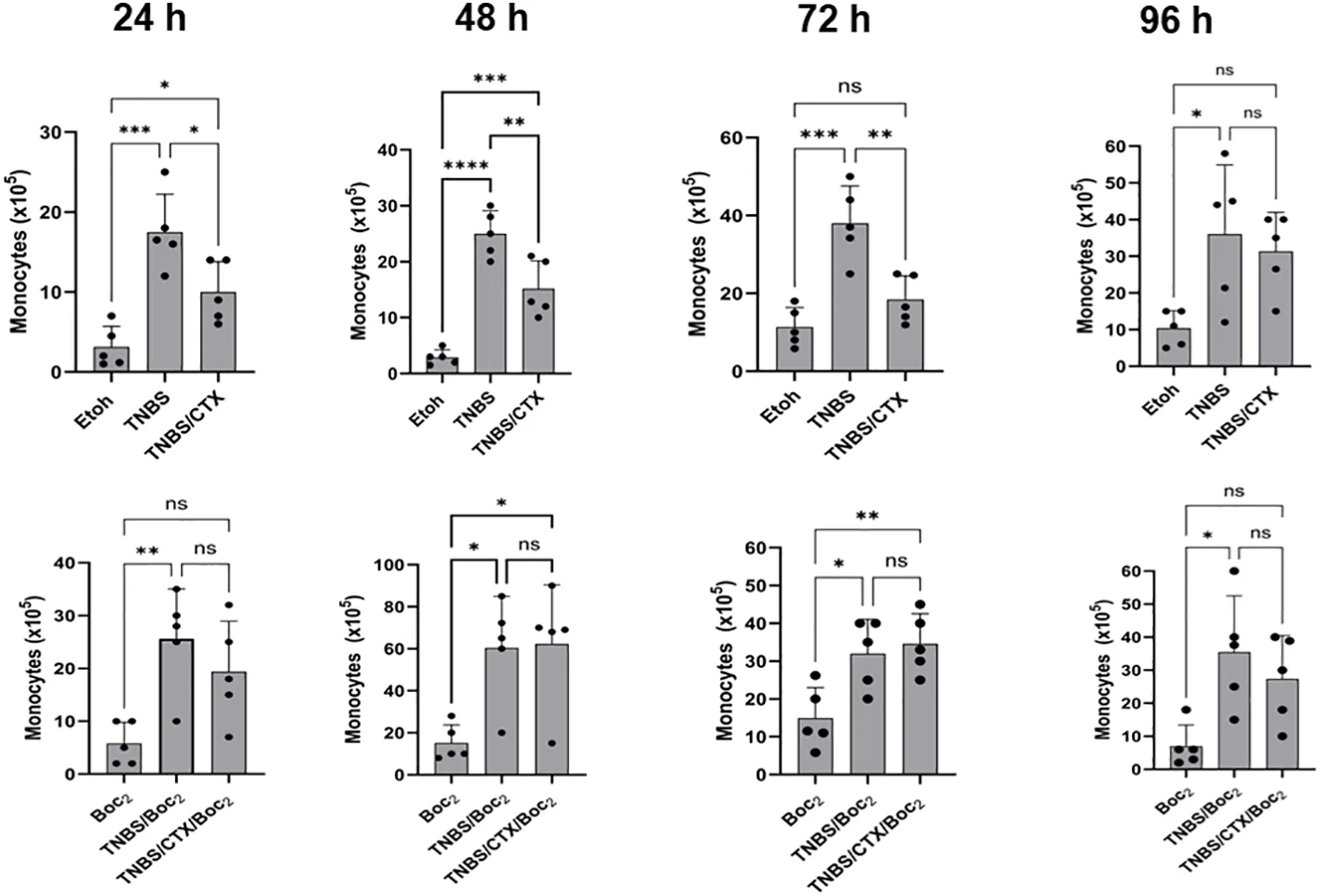
Effect of CTX on monocyte influx in BALB/c mice after acute colitis induction, treated or not with Boc_2_. Mice (n=5) received 100 µL of the TNBS solution intrarectal via. For induction control, a group of mice received 100 µL of 45% ethanol alone. Twelve hours after induction, the TNBS-mice groups were treated with CTX (0.045 mg/kg) or PBS (control) via i.p. injection. In a different set of experiments, groups of mice (n = 5) received intrarectal instillation of TNBS or Etoh. After 11 hours, they received or did not receive an intraperitoneal injection of the selective FPR antagonist Boc_2_ (0.5 mg/kg). After 1 hour, the animals received or did not receive CTX (0.045 mg/kg) via i.p. At 24, 48, 72, and 96h post-colitis induction, cell suspensions obtained from lamina propria were incubated with fluorescent antibodies: anti-CD45, anti-CD11b, and anti-CCR2, for the analysis of monocyte influx by flow cytometry. Results are expressed as the mean absolute number of positively labeled cells from individual animals/group ± SD. *p < 0.05; **p < 0.01; ***p < 0.001; ****p < 0.0001 ns, not significant.

Intestinal macrophages exert dual action according to the microenvironment; in healthy tissue, these cells participate in the maintenance of intestinal homeostasis, however in inflamed tissue, they can be drivers for the M1 phenotype secreting pro-inflammatory factors, exacerbating local inflammation, as observed in colitis (56). The **Figure 11** shows that TNBS-treated mice increased pro-inflammatory macrophages in comparison with the control-group (Etoh) at all time points analyzed. In contrast, this cell population was inhibited in TNBS-mice treated with CTX at 24, 48, and 72 h. At 96 h, no significant difference was observed between the TNBS and TNBS/CTX groups (**Figure 11**). In addition, CTX was not able to inhibit the pro-inflammatory macrophages in TNBS-mice previously treated with Boc_2_. In summary, our findings identify FPRs as key mediators of CTX-driven modulation in acute colitis.

**Figure 11:**
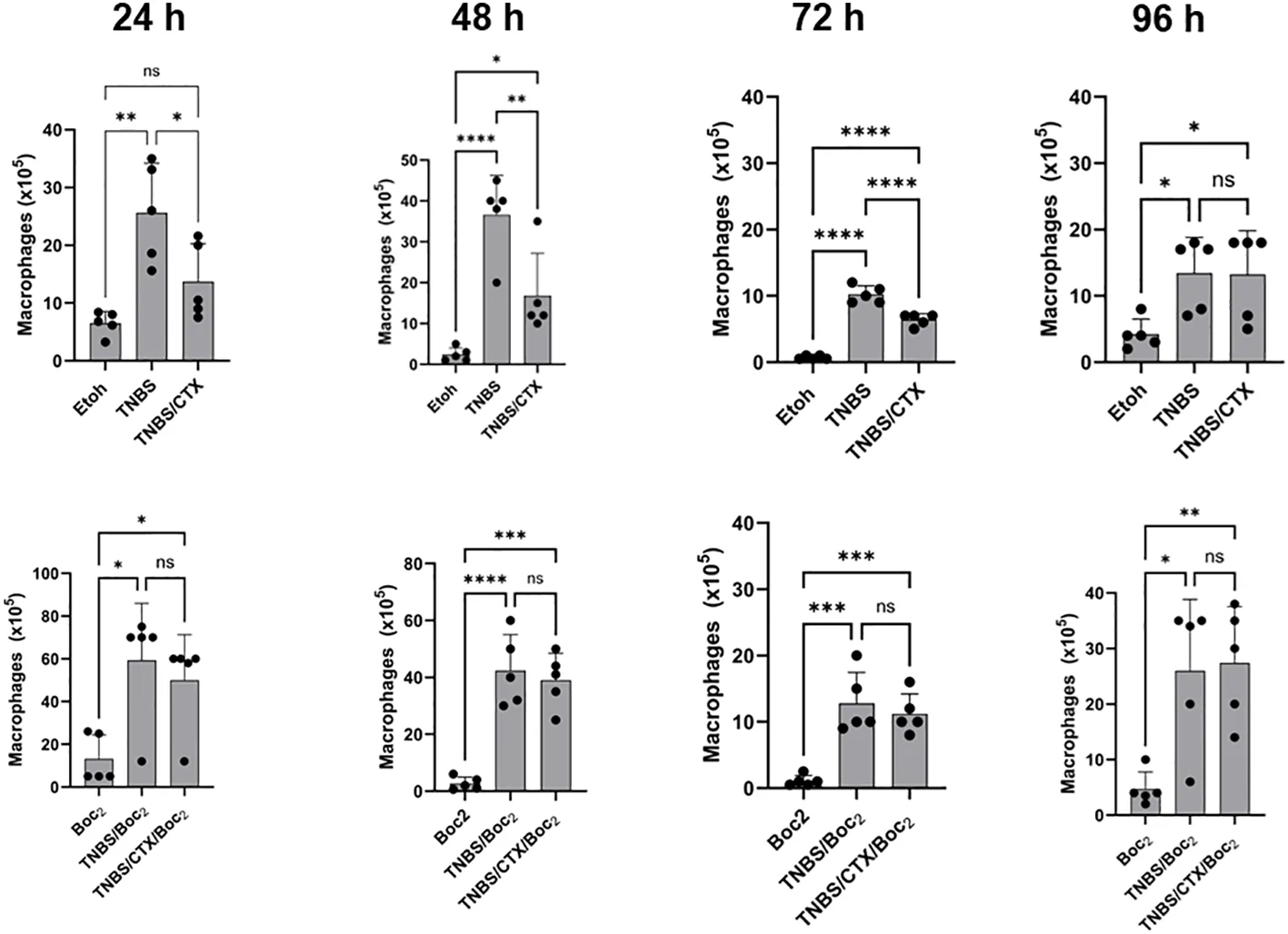
Effect of CTX on the macrophage population in BALB/c mice after colitis induction, treated or not with Boc_2_. Mice (n=5) received 100 µL of the TNBS solution intrarectal via. For induction control, a group of mice received 100 µL of 45% ethanol alone. Twelve hours after induction, the TNBS-mice groups were treated with CTX (0.045 mg/kg) or PBS (control) via i.p. injection. In a different set of experiments, groups of mice (n = 5) received intrarectal instillation of TNBS or Etoh. After 11 hours, they received or did not receive an i.p. injection of the selective FPR antagonist Boc_2_ (0.5 mg/kg). After 1 hour, the mice received or did not receive CTX (0.045 mg/kg) via i.p. At 24, 48, 72, and 96 hours after acute colitis induction, cell suspensions obtained from the lamina propria were incubated with fluorescent antibodies: anti-CD45, anti-CD11b, anti-MHC-II, and anti-CD68 for analysis of M1 macrophages by flow cytometry. Results are expressed as the mean absolute number of positively labeled cells/group ± SD. *p < 0.05; **p < 0.01; ***p < 0.001; ****p < 0.0001. ns, not significant.

### 3.10 CTX modulates ICAM-1 expression and IL-8 secretion in IFN-γ–activated Caco-2 cells

The activation of intestinal epithelial cells leads to increased ICAM-1 expression on the apical membrane, promoting neutrophil adhesion to the crypts and correlating with disease severity (61). In this context, to further investigate the impact of CTX on acute inflammation and the contribution of enterocytes to this process, we examined its effects on Caco-2 cell activation. In addition, IL-8 secreted by enterocyte plays a central role in directing neutrophil migration to sites of inflammation (62). Therefore, we analyzed the ICAM-1 expression in Caco-2 cells incubated with IFN-γ *in vitro*. As showed in **Figure 12A**, an enhancement of ICAM-1 expression was induced in Caco-2 cultures incubated with IFN-γ compared with the culture maintained in medium (control). On the other hand, CTX was able to inhibit IFN-γ-induced ICAM-1 expression. Additionally, ICAM-1 expression in cells treated with CTX alone was comparable to that observed in the control group. Our results also showed that IFN-γ significantly upregulated IL-8 production in Caco-2 cultures compared with cells in the presence of medium or CTX. Notably, IFN-γ/CTX group resulted in a marked reduction of IL-8 levels (**Figure 12B**).

**Figure 12:**
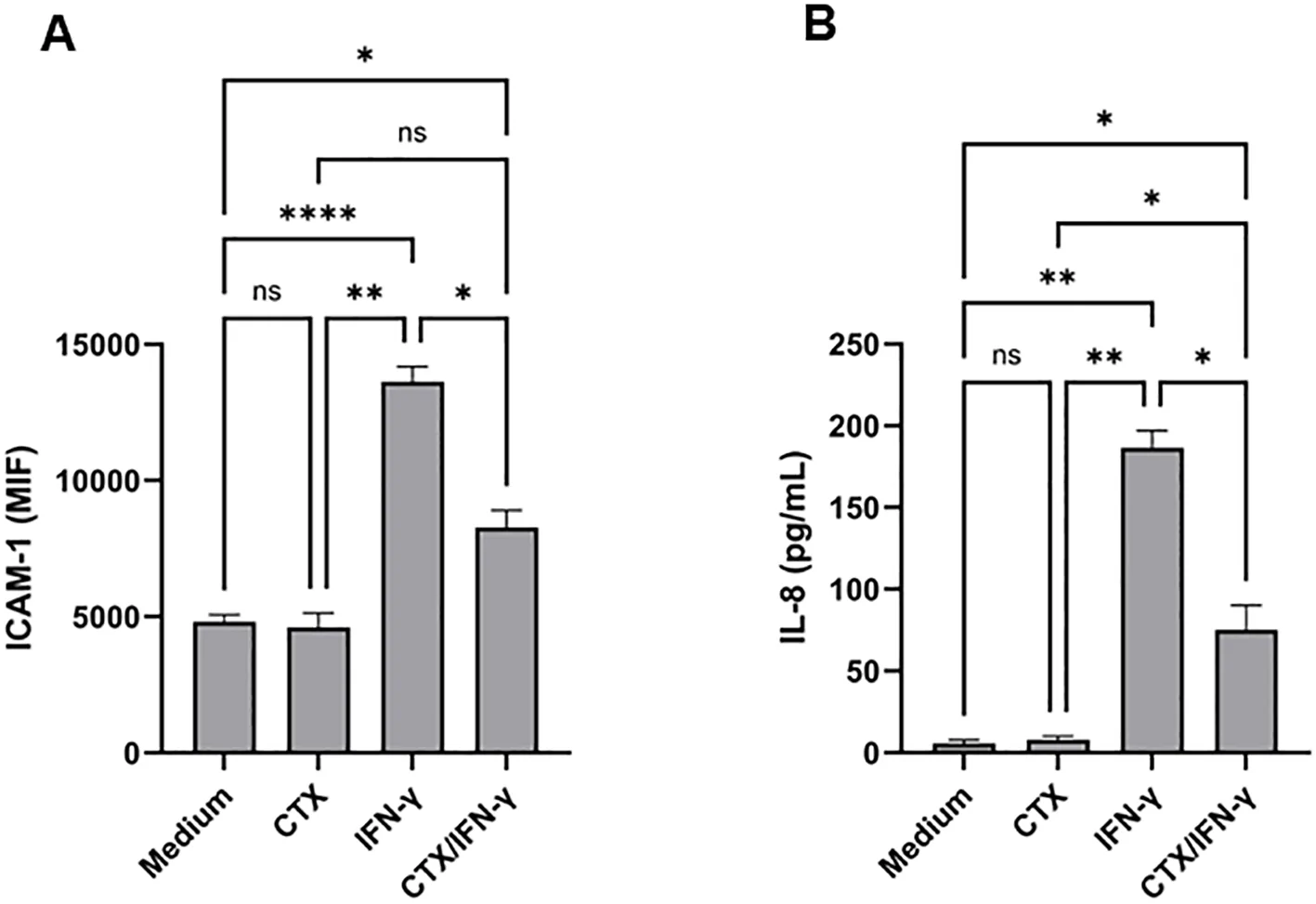
**(A)** ICAM-1 expression and **(B)** IL-8 production in differentiated Caco-2 cells *in vitro* incubated with IFN-γ in the presence or absence of CTX. Caco-2 cells were stimulated with IFN-γ (40 ng/mL), CTX (250 ng/mL), or IFN-γ/CTX (40 ng/mL + 250 ng/mL) for 18 hours. Subsequently, cell supernatants were collected and used to determine IL-8 levels by ELISA. Cells were then stained with anti-ICAM-1-FITC antibody and analyzed by flow cytometry. Results are expressed as the mean fluorescence intensity (MFI) of triplicate samples ± SD. Representative results from three independent experiments are shown, *p < 0.05; **p < 0.01; ***p < 0.001, ****p < 0.0001; ns, not significant.

### 3.12 CTX inhibits human neutrophil and monocyte migration induced by IFN-γ-stimulated enterocytes

A hallmark of intestinal inflammation is the increased recruitment of neutrophils, followed by monocytes, into the intestinal mucosa (61). Furthermore, since epithelial cells, when activated, participate in the migration of inflammatory cells in the development of colitis, we sought to figure out the migration of human neutrophil and monocyte in a transwell system using supernatants from pre-stimulated Caco-2 cells. For this, supernatants from Caco-2 cells incubated with IFN-γ promoted neutrophil and monocyte migration compared with control culture (**Figures 13A, B**). In contrast, supernatants from Caco-2 cells treated with IFN-γ/CTX induced a reduction in neutrophil and monocyte migration compared with IFN-γ alone. Supernatants from Caco-2 cells treated with CTX alone did not significantly affect the migration of either cell type.

**Figure 13:**
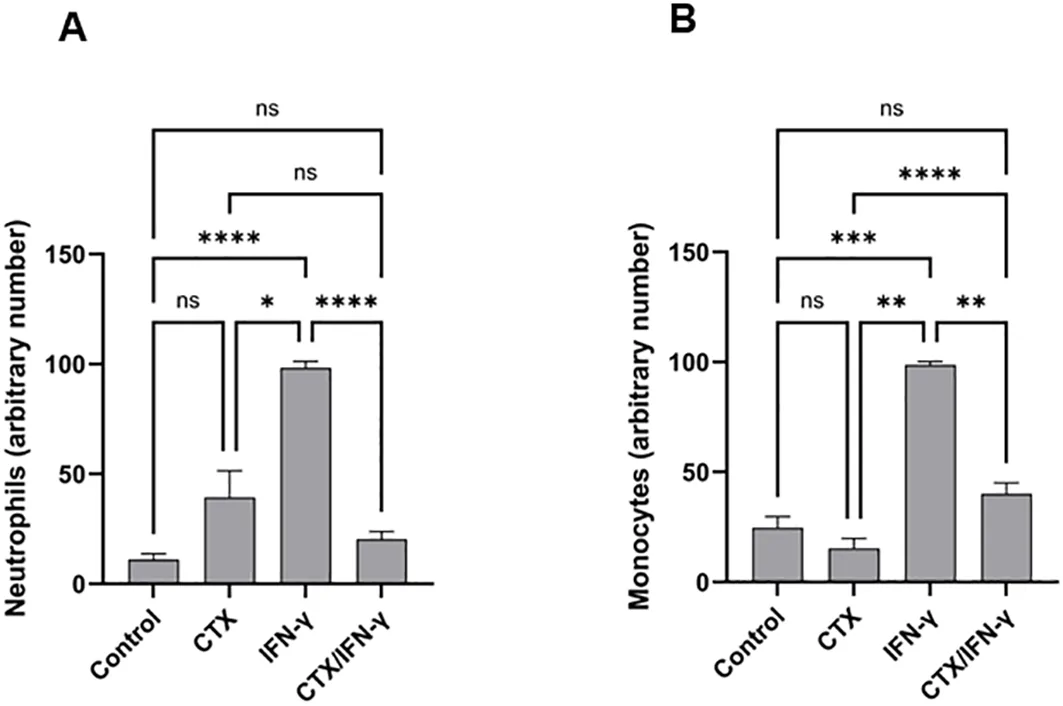
Effect of CTX on *in vitro* human neutrophil and monocyte migration induced by supernatants from IFN-γ-stimulated Caco-2 cells. Differentiated Caco-2 cells (17–18 days) were incubated for 18 hours with IFN-γ (40 ng/mL), CTX (250 ng/mL), or IFN-γ/CTX (40 ng/mL + 250 ng/mL). Supernatants were then collected and used in a transwell migration assay. Results are expressed as the mean total number of migrated cells in triplicate ± SD. Representative results from three independent experiments are shown, *p < 0.05; **p < 0.01; ***p < 0.001; ****p < 0.0001; ns, not significant.

## 4 Discussion

Despite advances in therapies for intestinal inflammation, long-term toxicity and therapeutic resistance present ongoing challenges (63–65), driving the search for alternative approaches. Expanding on previous evidence that CTX ameliorates acute TNBS-induced colitis (35), the present study explored its impact on the establishment of intestinal inflammation and set out to elucidate the underlying mechanisms.

In *in vitro* experiments, we verified that CTX was able to modulate intestinal barrier integrity in IFN-γ–stimulated Caco-2 cells (10, 66–70). In agreement with our results, Roselli et al. (71) also demonstrated the modulatory capacity of the prebiotic Bimuno GOS on DSS-induced inflammatory responses in Caco-2 cells, significantly inhibited the production of pro-inflammatory cytokines (IL-8, TNF-α, and IL-6). In addition, the prebiotic mitigated DSS-induced damage to intercellular junctions in *in vitro* cultures.

Inflammatory mediators released by activated mucosal immune cells are known to alter both the morphology and function of the intestinal epithelial layer. In particular, IFN-γ modulates the expression of molecules involved in cell–cell and cell–matrix interactions (72, 73) and its production is increased in patients with IBD (74, 75). Our results corroborate previous reports, as IFN-γ stimulation resulted in reduced expression of the tight junction protein ZO-1, leading to decreased transepithelial electrical resistance and impairment of epithelial barrier integrity. Conversely, CTX effectively CTX exerted direct protective and anti-inflammatory effects by inhibiting IFN-γ–induced ICAM-1 expression and IL-8 secretion in Caco-2 cells, which subsequently reduced the recruitment of human neutrophils and monocytes in transwell migration assays. Collectively, these findings indicate that CTX acts directly on the intestinal epithelium and, consequently, modulates inflammatory cell migration in response to an stimulus such as IFN-γ.

Among the studies describing the effects of CTX on leukocyte influx in response to inflammatory stimuli, Nunes et al. (48) demonstrated that CTX modulates leukocyte–endothelial interactions in the cremaster muscle of carrageenan-injected mice. Treatment with CTX resulted in increased leukocyte rolling and reduced leukocyte adhesion to the vascular endothelium, as assessed by intravital microscopy. Similarly, Zambelli et al. (76) reported that *in vivo* administration of CTX altered the expression of cell adhesion molecules, leading to a reduction in the number of circulating leukocytes in peripheral blood. More recently, Kato et al. (28) demonstrated that CTX exerts a direct effect on endothelial cell function (t.End.1 cells) and inhibits key angiogenic processes, including cell adhesion, proliferation, and migration, in an *in vitro* model. Considering these findings and in continuation of our objective to investigate the mechanisms underlying the modulatory effects of CTX on acute intestinal inflammation, we reproduced the TNBS-induced IBD model through intrarectal instillation in BALB/c mice and evaluated the immunomodulatory potential of CTX, as previously described by Almeida and col. (35).

Formyl peptide receptors (FPRs) are G protein–coupled receptors involved in inflammation, tissue homeostasis, and the resolution of mucosal inflammatory responses (77–79). The repertoire of FPR ligands includes not only microbial N-formyl peptides but also non-formylated peptides of microbial or host origin, as well as synthetic small molecules and eicosanoids (79). FPR1 was the first member of the FPR family to be identified and displays higher affinity for bacterial formylated peptides compared to FPR2. In contrast, FPR2 recognizes a broader range of ligands, including amyloid peptides, antimicrobial peptides, and lipid mediators. FPRs are expressed by cells of the innate immune system, such as neutrophils, monocytes, and macrophages, as well as by epithelial cells, endothelial cells, neurons, and hepatocytes (78, 79). Among the endogenous ligands of FPR2, annexin A1 (ANXA1) and lipoxin A4 (LXA4) exert potent anti-inflammatory effects and play key roles in the resolution of inflammation, as demonstrated in *in vivo* studies (78–80).

Regarding CTX-mediated immunomodulation, several studies have reported the involvement of LXA4 in the effects of this toxin on neutrophils, macrophages, and lymphocytes via FPR signaling pathways (24, 48, 58, 59, 80). These studies demonstrated that the selective FPR antagonist Boc_2_ effectively reverses the inhibitory action of CTX on carrageenan-induced leukocyte migration (48), macrophage phagocytic activity (80, 81), and T lymphocyte proliferation, as well as antibody production by B lymphocytes (59). Furthermore, the involvement of FPR2 has been demonstrated in the modulation exerted by CTX on dendritic cell functional activity *in vitro* (82). Consistently, treatment with Boc_2_ abolished the inhibitory effects on endothelial cell adhesion and proliferation induced by supernatants from macrophages preincubated with CTX (26).

Based on these observations, we evaluated FPR involvement in CTX-mediated protection against TNBS-induced colitis by using Boc_2_, a FPR antagonist. Boc_2_ administration fully abrogated the protective effect of CTX on clinical scores, colonic histology, and goblet cell in TNBS-induced colitis. Additionally, Boc_2_ blocked the modulation exerted by CTX on neutrophil (Ly6G^+^CD11b^+^CD18^+^), monocyte (CD11b^+^CCR2^+^), and M1 (CD11b+MHCII+CD68+) macrophage infiltration into the lamina propria of TNBS-mice. Taken together, these data indicate a central role of FPR signaling in the protective properties of CTX in acute colitis.

Finally, within the complex network of factors involved in the development and establishment of IBDs, dysfunctional interactions between the intestinal microbiota and the mucosal immune system can lead to a breakdown of intestinal immune tolerance, resulting in an exaggerated immune response. Dysbiosis is a hallmark of IBD, characterized by reduced microbial diversity. In this context, several studies have demonstrated that modulation of the intestinal microbiota contributes to symptom improvement in ulcerative colitis (UC) (57, 83). Moreover, evidence supports the efficacy of probiotic and prebiotic supplementation in positively modulating gut microbiota composition, promoting mucosal restitution, which is essential for intestinal health, immune homeostasis, and maintenance of remission in patients with UC (45, 83, 84). In addition to microbial factors, genetic susceptibility also plays a significant role in IBD pathogenesis. Genome-wide association studies, next-generation sequencing, and related analyses have identified approximately 30 genetic loci shared between Crohn’s disease and ulcerative colitis, further highlighting the multifactorial nature of these disorders (56).

Our *in vitro* findings demonstrating that CTX preserves epithelial barrier integrity are strongly supported by our new *in vivo* microbiota analysis. Acute inflammation induced by TNBS damages the intestinal epithelium and creates an environment conducive to dysbiosis, as shown by the dramatic increase in Proteobacteria, primarily Escherichia. This expansion of pathobionts is a common feature in intestinal inflammation and is likely facilitated by compromised barrier function. Therefore, the ability of CTX to maintain ZO-1 expression and transepithelial resistance is a key mechanism that likely limits the expansion of these opportunistic bacteria, thereby helping restore microbial homeostasis.

In summary, our *in vivo* and *in vitro* data clarify the effect of CTX in the initial events of acute colitis in mice, highlighting its potential immunomodulatory properties. Based on this rationale, future investigations will assess the capacity of the CTX to attenuate the chronic model of intestinal inflammation, supporting the development of novel therapeutic strategies for IBD.

## 5 Conclusions

The protective effect of CTX preserved the integrity barriers and then, prevented the pro-inflammatory cell recruitment and consequent establishment of the tissue inflammation. Importantly, our data link this entire protective cascade to FPR pathway. The dysbiotic state induced by TNBS at 24-hours’ time point, characterized by a high abundance of Proteobacteria, generates a significant load of bacteria-derived formyl peptides, leading to hyperactivation of FPRs and sustained immune cell recruitment. The finding that the protective effects of CTX were abolished by the FPR antagonist Boc_2_, along with our observation that CTX partially restores the microbiome, suggests a dual mechanism. CTX not only modulates the host response via FPR pathway but also reduces the source of the pro-inflammatory bacterial ligands that activate this pathway. Therefore, the CTX exerts a protective effect in an acute intestinal inflammation murine model by preserving and modulating immune responses through FPR-mediated pathway.

## 6 Limitations

This study has certain limitations that should be acknowledged. First, although the acute TNBS-induced colitis model effectively recapitulates key histopathological features of Crohn’s disease, it represents an acute inflammatory response rather than the chronic, relapsing-remitting nature of human IBD. Second, while the FPR antagonist Boc_2_ was used to investigate receptor involvement, pharmacological blockade may carry off-target effects; future studies utilizing FPR knockout mouse models are required to definitively validate these receptor-specific mechanisms.

## Data Availability

The original contributions presented in the study are included in the article/**Supplementary Material**. Further inquiries can be directed to the corresponding authors.
