# Supplementary material for: Crotoxin from Crotalus durissus terrificus impairs the establishment of chemically induced colitis in mice associated with formyl peptide receptor signaling
Source: Front Immunol. 2026 Sep 16;17:1886884. doi: 10.3389/fimmu.2026.1886884 (PMC13623570; doi:10.3389/fimmu.2026.1886884)
Supplement: Supplementary file 1 [file Supplementaryfile1.docx]

Supplementary Material

**Figure S1: Experimental *in vivo* protocol for TNBS-induced acute colitis.** Acute colitis was induced by intrarectal instillation of 2 mg of TNBS dissolved in an equal volume of 45% ethanol in mice (n = 5) anesthetized with a physiological solution containing ketamine (90 mg/kg) and xylazine (10 mg/kg). Mice received 100 µL of the TNBS solution administered slowly via a 3.5 F catheter attached to a 1 mL syringe. In the induction control group, mice received 100 µL of 45% ethanol alone. Twelve hours after induction, TNBS-treated mice were administered CTX (0.045 mg/kg) or saline (control) via intraperitoneal injection.

**Table 1.S2: Antibodies used for labeling murine neutrophils.**

| Antibody | Clone | Fluorochrome | Manufacturers | Dilution | Expiration |
| --- | --- | --- | --- | --- | --- |
| anti-CD45 | 30F1 | PercpCy 5.5 | Biolegend | 1:100 | 2026.05.27 |
| anti-CD11b | M1/70 | PeCy7 | Biolegend | 1:100 | 2026.02.20 |
| anti-Ly6G | 1A8 | FITC | Biolegend | 1:100 | 2026.08.10 |
| anti-CD18 | M18/2 | PE | Biolegend | 1:100 | 2026.07.05 |

**Table 2. S3: Antibodies used for labeling murine monocytes and macrophages.**

| **Antibody** | **Clone** | **Fluorochrome** | **Manufacturers** | **Dilution** | **Expiration** |
| --- | --- | --- | --- | --- | --- |
| anti-CD45 | 30-F11 | PercpCy 5.5 | Biolegend | 1:100 | 05/2026 |
| anti-CD11b | M1/70 | BV421 | Biolegend | 1:100 | 06/2026 |
| anti-MHCII | MS/114.15.2 | PE | Biolegend | 1:200 | 07/2026 |
| anti-CCR2 | SA203G11 | AF647 | Biolegend | 1:80 | 02/2026 |
| anti-CD68 | FA-11 | PECY7 | Biolegend | 1:200 | 02/2026 |

**Figure S4 - Analysis strategy applied to cells isolated from the lamina propria of different groups of mice.** (A) Determination of total cells based on FSC-A vs SSC-A; (B) exclusion of debris and cell aggregates; (C) gating strategy to select viable cells (FVS-510/anti-CD45); (D) fluorescence minus one (FMO) control for neutrophils using SSC-A/anti-Ly6G and (E) MAC-1 (anti-CD11b/CD18) to define the neutrophil population; FMO control to determine the monocyte population using (F) anti-CD11b/anti-CCR2 and (G) anti-CD11b/anti-MHCII/anti-CD68 to define the macrophage population.

**Table 3. S5: Antibodies used for labeling neutrophils and monocytes for cell sorting.**

| **Antibody** | **Clone** | **Fluorochrome** | **Manufacturers** | **Dilution** | **Expiration** |
| --- | --- | --- | --- | --- | --- |
| anti-CD14 | M5E2 | FITC | Biolegend | 1:100 | 08/2026 |
| anti-CD16 | 3G8 | APC | Biolegend | 1:100 | 08/2026 |
| anti-CD11b | 1M2 | PE | Biolegend | 1:100 | 07/2026 |
